# Daily stress, and mental health of professional degree graduate students in Chinese traditional medicine universities: the mediating role of learning career adaptation

**DOI:** 10.1186/s12909-023-04614-5

**Published:** 2023-09-03

**Authors:** Ling Li, Mingling Zhu, Anling Yao, Jialu Yang, Lili Yang

**Affiliations:** 1 School of Nursing, Zhejiang Shuren University, 8 Shuren Road, Hangzhou, Zhejiang Province 310053 China; 2 School of Nursing, Zhejiang Chinese Medical University, 548 Bin-wen Road, Hangzhou, Zhejiang Province 310053 China

**Keywords:** Professional degree graduate students, Daily stress, Learning career adaptation, Mental health, Mediating effect

## Abstract

**Background:**

With the expansion of professional degree graduate students’ enrollment in China education, the mental health of these professional degree graduate students in medical-related majors who are under pressure of study, scientific research, clinical practice, and employment should not be ignored. What is the mental health level of these graduate students under the effect of learning career adaptation (internal resources) in the face of daily stress (external factors)? The purpose of this study is to discuss the relationship between these variables, and the mediating role of learning career adaptation of professional degree graduate students in traditional Chinese medicine colleges, and universities, to provide a theoretical basis for improving the learning career adaptation of students, and improving the level of mental health.

**Methods:**

A cross-sectional survey was conducted among 1593 professional degree graduate students majoring in clinical medicine, traditional Chinese medicine, and nursing in five traditional Chinese medicine universities. Finally, 660 questionnaires were returned, with a recovery rate of 41.43%. The scores of daily stress, learning career adaptation, and mental health were measured by Daily Stressors Scale for graduate students, graduate-students learning career adaptation scale, and General Mental Health Questionnaire (GHQ-20). Descriptive statistics were used to analyze the status quo of daily stress, learning career adaptation, and mental health. Pearson correlation analysis were used to analyze the relationship between them. we undertake analyses using structural equation modeling to construct the latent variable path model of daily stress, learning career adaptation on mental health. The significance level of the mediating effect was tested by the non-parametric percentile bootstrap method.

**Results:**

The scores of mental health, daily stress, and learning career adaptation were 50.56 ± 10.80, 35.12 ± 19.55, and 67.13 ± 7.48 respectively. Daily stress was negatively correlated with the three dimensions of learning career adaptation: career confidence, focus on his career, and career control (P < 0.01). Daily stress was positively correlated with depression and anxiety (P < 0.01). Self-affirmation, depression, and anxiety were negatively correlated with career confidence, focus on his career, and career control (P < 0.05). Learning career adaptation plays a partial mediating role between daily stress, and mental health (p < 0.001), with an intermediate effect value of 0.127, representing 28.54% of the total effect.

**Conclusions:**

Mental health, learning career adaption of medical-related professional degree graduate students in traditional Chinese medical universities were at a moderate degree, and an upper-middle level respectively, while daily stress is to a lesser extent. Learning career adaptation mediates the relationship between daily stress, and mental health partially. To some extent, it can buffer the impact of daily stress on mental health, especially anxiety. The educational administrator could take various measures to improve the mental health of professional degree graduate students. It can also enhance their learning career adaptation from the perspective of individuals, and organizations to improve their mental health.

**Supplementary Information:**

The online version contains supplementary material available at 10.1186/s12909-023-04614-5.

## Background

Professional degree graduate students, commonly known as “applied graduate students”, can be divided into ordinary graduate students, and special types of graduate students (professional degree graduate students) according to different majors, and uses. As China’s economy and society entered a stage of high-quality development, the Ministry of Education decided to introduce full-time training for most professional master’s degree from 2009 following the needs of China’s initiative to serve the construction of an innovative country. In 2011, we continued to implement the policy of changing the focus of postgraduate education from academic talents to applied talents. Professional degree graduate students’ education is aimed at cultivating high-level applied talents combined with theory, and practice for a certain professional background.

The education of “professional degree postgraduates” in China involves master’s degrees (39 kinds) and doctor’s degrees (5 kinds), basically covering the main fields of national economic, and social development. There are 509 training units with the right to confer graduate professional degrees, and more than 1 million professional degree postgraduates have been enrolled. China is the second largest country for graduate education after the United States. Based on the national enrollment of clinical medicine professional degrees graduate students in 2009, the Ministry of Education added the professional master’s degree setting of finance, applied statistics, taxation, pharmacy, nursing, traditional Chinese medicine, clinical medicine (including integrated traditional Chinese medicine, and Western medicine) in January 2010 [1]. Many traditional Chinese medicine colleges and universities have recruited postgraduates majoring in clinical medicine, traditional Chinese medicine, and nursing in addition to Western medicine universities to meet the increasing medical service demands of Chinese people. By 2025, the Academic Degrees Committee of the State Council will expand the amount of professional master’s degree students to about two-thirds of the total enrollment [2]. For such a large-scale enrollment of professional degree postgraduate students, the teaching training, management, and maintenance of physical, and mental health are all the problems that the educational managers should pay attention to.

The researchers have been engaged in higher education of medical-related majors for many years, focusing on the mental health of medical-related students, especially how to deal with the triple pressure of teaching, clinical practice, and scientific research of medical-related professional degree postgraduates. According to the notice of the Academic Degrees Committee of The State Council, the training of postgraduates with professional degree postgraduates in clinical medicine, and traditional Chinese medicine should be connected with the standardized training of residents [3]. The duration of standardized training is generally 3 years, that is, the last year will be arranged to work as a general resident or corresponding hospital management after two years of in-depth study and mastery of clinical skills, and theoretical knowledge. On the one hand, the training of professional degree Master of Nursing adopts the combination of teachers in the school of nursing, and tutors (or associate tutors) in the clinical hospital, and the steering group carries out collective training for professional degree Master of Nursing. The training mode is also based on clinical practice, supplemented by certain courses, and scientific research training. Therefore, professional degree postgraduate students in medical-related majors are required to strengthen their clinical practice, and the three-year learning career needs to complete theoretical courses, clinical practice, dissertation, and other learning tasks. The training and education of traditional Chinese medicine colleges and universities often combine the characteristics of traditional Chinese medicine, that is, to add traditional Chinese medicine related courses to the academic content, resulting in greater pressure. While the outbreak of COVID-19 has brought significant changes, and inconvenience to students in 2020, including online courses, and social isolation. As medical-related students, they would undertake more volunteer services, inconvenience in school study, and clinical practice, and the struggle for job hunting, which all lead to a high incidence of anxiety, and depression. A study found that the rates of mild, moderate, and severe depression among graduate students were 21.99%, 10.48%, and 1.4%, and the proportion of anxiety was 1.56%, 4.65%, and 14.69%, respectively [4]. Graduate students are also six times more likely to be depressed than the general population [5]. Some studies have found an increase in emotional distress of college students in China, and India, and a decline in mental health in the UK before, and during the COVID-19 outbreak [6–8], and a North American study found that occupational therapy masters students reported some academic related stress, long hours of study, examinations, and long lectures [9]. Moreover, the level of academic pressure has increased even more after the COVID-19 pandemic. In addition, graduate students may experience academic bullying, sexual assault, or violence from peers or authorities (supervisors, professors, etc. [10] As for coping with stress, some students choose to limit food intake, choose unhealthy eating, or overeating, 67.55% of the students believed that physical exercise could effectively relieve stress, especially the decompression effect of taekwondo. In addition, social participation or talking with family, and friends could also be considered as a coping behavior [9]. It has been suggested that depression in graduate students affects research motivation, concentration, memory, creativity, and participation in research [11], which are not addressed timely, and effectively, will affect their studies, and personal lives, leading to other extreme behavior [12]. Graduate students may even consider dropping out more frequently than other medical students, and residents [13]. For the treatment of mental health, mindfulness interventions [14], cognitive behavioral interventions [15], psycho-educational interventions [16], skill-oriented interventions including supervision skills [17], educational/individualized feedback interventions, and universal mental health prevention programs (containing relaxation, environment-based interventions, or stress reduction interventions [18]) have been shown to be effective to some extent.

The incidence of students’ mental health problems is strongly related to their academic role disorders [19]. Findings also suggested that the proportion of mental health problems caused by academic role impairment is much higher than that of physical problems [19]. The interventions to improve students’ mental health enhance students’ academic role performance significantly compared with successful interventions to improve students’ physical health [19]. The Ideological and Political Office of the Ministry of Education issued the ‘Basic construction standard of mental health education for students in ordinary colleges and universities’ in 2011, which further standardized the work of mental health education in colleges and universities and also obtained a certain effect. However, there are still some problems in the mental health service of graduate students, such as one-sided education content, single teaching form, lack of sound psychological education evaluation system, lack of staff, etc. The mental health of graduate students lags behind that of undergraduates in reality [20].

### Relationship between daily stress, and mental health

Life events are a common psychosocial stressor affecting physical, and mental health [21]. Daily exposure to psychosocial stressors can affect micro vasoconstrictor function adversely, regardless of the perceived severity of the stressor or the emotional outcome of the disclosure [22]. Data from several longitudinal studies suggested that people exposed to daily stress have increased cardiovascular responsiveness to stress-induced sympathetic activity [23], with daily stress being able to predict long-term health, and well-being reactivity [24]. Studies have shown that daily stress processes play an essential role in predicting mental, and physical health impairments [24–26] and increased mortality [27]. Daily stressors also have a negative relationship with positive mental health [28]. Negative life events are often associated with debilitating mental health problems, such as anxiety, and depression [29], depression in particular [30]. Daily stress, therefore, is a better predictor of physical, and mental health than infrequent major life events [31].

### Relationship between daily stress, and learning career adaptation

Stress is a part of life and an unavoidable topic [32]. Stress management is the process of change that individuals use to reduce stress [33]. Career adaptation refers to the ability of individuals to cope with the change of occupational role, and to find a balance between occupational role, and the pressure of the working environment [34]. One study found that career adaptation was associated with adaptive variables such as cognitive ability, big five traits, self-esteem, etc., adaptive responses such as career exploration, and self-efficacy, and adaptive outcomes such as job/career/school satisfaction, job stress, employability, etc. [35] A study of nursing students before internship found that perceived stress before internship was negatively associated with pre-internship career adaptation, the correlation coefficient was − 0.69 [36].

### Relationship between learning career adaptation and mental health

Career adaptation refers to the ability of individuals to adapt, cope with unpredictable events in their career, and maintain a balance in their professional roles [37]. Individuals who have difficulty adapting to foreseeable and unforeseen career-related challenges may experience anxiety, and other negative mental health outcomes [38]. Zhou believed that work adaptability is an important predictor of occupational mental health, and the higher the level of work adaptability, the better the mental health of individuals, and the better the career success [39]. It has been suggested that career adaptation of adolescents can play a protective role in reducing career decision-making difficulties, and mental health problems [40].

### The mediating role of learning career adaptation

With the rapid development of society, and the economy, a necessary condition for success is to learn how to adapt to a changing world. Occupational adaptation can help individuals deal with their occupational roles smoothly when adapting to changes, and maintain their ability to balance their occupational roles [37]. This can help individuals see the possibility of unexpected changes, and use those to recover from unforeseen outcomes [41].

The higher the occupational adaptation of individuals, the more psychosocial resources they need, which can help them to deal with tasks, transitions, and traumatic events successfully in their careers [31]. Career adaptation also plays a mediating role in the relationship between employment stress, and suicide ideation [11], which could be seen as a strong predictor of mental health problems [42, 43]. Career adaptation plays a partially mediating role in the understanding of the impact of social support on mental health among graduate students [44]. However, previous studies have focused on career adaptation, but the effect of learning career adaptation on the mental health of professional degree postgraduates under daily stress is not clear.

**Fig. 1:**
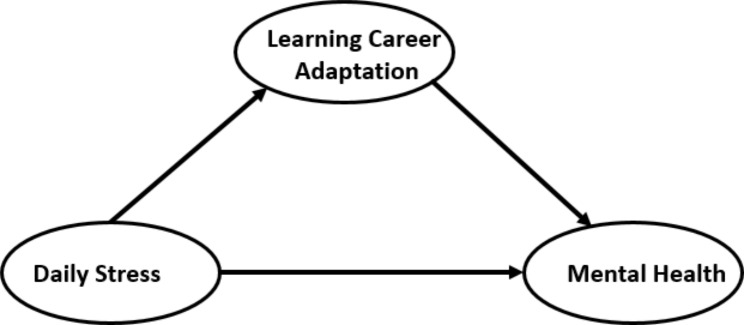
Hypothetical model

Based on the above-mentioned literature reviews, this study puts forward the following hypotheses: First, daily stress is related to the mental health of professional degree graduate students in medical-related majors (H1). Second, Daily stress is correlated with learning career adaptation of professional degree graduate students in medical-related majors (H2). Third, learning career adaptation is correlated with the mental health of professional degree graduate students in medical-related majors (H3). Finally, learning career adaptation plays a mediating role in the relationship between daily stress, and mental health. (H4). The hypothetical figure is shown in Fig. 1.

Considering the importance of daily stress, and learning career adaptation on the mental health of medical-related professional degree graduate students, this study investigated the daily stress, learning career adaptation, and mental health levels of professional degree graduate students in traditional Chinese medicine universities, aiming to explore the relationship between the three variables, and the mediating effect of learning career adaptation between the other two variables, put forward some suggestions for improving the learning career adaptation, and promoting their mental health of professional degree graduate students, to provide empirical support for educational managers to take effective, and comprehensive measures to improve the mental health of medical-related professional degree graduate students from the perspective of learning career adaptation, which is an internal psychological resource.

## Materials and methods

This was a cross-sectional study conducted utilizing an online survey questionnaire between July and December 2020.

### Questionnaire design and distribution

The questionnaire was designed, and distributed to collect the impact of the COVID-19 pandemic in China on the learning career adaptation, and mental health of professional degree graduate students in traditional Chinese medicine universities. The professional degree graduate students in the school of nursing contacted their undergraduate classmates (currently studying for master’s degrees in different universities) to participate in the questionnaire survey.

### Participants

According to the calculation method of sample size in observational studies, the sample size should be 5 ~ 10 times the number of independent variables [45]. A total of 68 independent variables were included in this study, so the sample size was 136~680 cases. Considering the invalid questionnaire, the sample size was expanded by 10%, and the required sample size was 150. However, the minimum sample size based on structural equation modeling should be 10 times larger than the estimated number of paths in the model [46], so the minimum sample size in this study was 170 cases. Considering the deformation rate of 10%, the appropriate sample size in this study was 180 cases. 635 professional degree graduate students majoring in clinical medicine, traditional Chinese medicine, and nursing from the Zhejiang University of Chinese Medicine, Fujian University of Chinese Medicine, Hubei University of Chinese Medicine, Guiyang University of Chinese Medicine, and the Nanjing University of Chinese Medicine were recruited as the survey subjects. Inclusion criteria were (1) full-time professional degree graduate students; (2) willing to participate in the survey;3) having certain listening, speaking, reading, and writing skills. Exclusion criteria were (1) part-time professional degree graduate students; (2) Master of Science degree students;3) unwilling to participate in the survey. (See Table 1)

### Data collection

In this study, researchers distributed questionnaires on the Questionnaire online platform(https://www.wenjuan.com/edit/survey/5dcd0b1a92beb5106d83e0b4?scene=survey#brand) from July 2020 to December 2020, See Additional file 1 for details. On the home page, the purpose, significance, and projected completion time of the survey were described. Using convenience sampling, and selected samples based on informed consent. Respondents fill in the questionnaire independently, which takes about 15–20 min.

### Survey tools

#### General Health Questionnaire (GHQ-20)

The questionnaire was translated, and revised by Li Hong and Mei JinRong based on GHQ-30 [47]. It consists of three dimensions: self-affirmation (9 items), depression (6 items), and anxiety (5 items), with each entry using the Likert scale five score method, with points 1–5 representing " completely does not comply, less complies, uncertain, mostly complies, and fully complies.“ This set-up self-affirms dimension reverse transformation, and generates self-denial dimensions. The scores of the three dimensions were summed to form the total negative mental health score. A higher score meant a lower total mental health level. The internal consistency of the questionnaire ranged from 0.60 to 0.83, and the correlation validity of its subscales ranged from 0.32 to 0.67, thus fulfilling all psychological measurement requirements. The Cronbach’s α coefficient of this study is 0.854.

#### Graduate students learning career adaptation scale

This study used the career adaptation questionnaire for graduate students based on Zhao [48], revised by Hua [49]. The questionnaire comprised four dimensions: focus on his career, career curiosity, career confidence, and career control, totaling 17 questions. It likewise used the Likert 5-point scale with 1 representing “completely inconsistent”, 2 representing “inconsistent”, 3 representing “uncertain”, 4 representing “consistent”, and 5 representing “completely consistent”. The test results show that the questionnaire exhibited good reliability, and validity. The internal consistency reliability coefficient of the total scale is 0.874, and the internal consistency reliability of the four subscales is at 0.667, 0.658, 0.794, and 0.68, respectively. The Cronbach’s α coefficient of this study is 0.820.

#### Daily stressors scale for graduate students

The daily stressors scale for graduate students compiled by Huang [50] was also adopted by this study. It consists of 31 items on the scale, including six dimensions: economy, study, employment, change, love and sex, and social. The scale also adopts five grades: no impact, mild, moderate, severe, and extremely severe, represented by 0, 1, 2, 3, and 4 points on a Likert scale, respectively. The internal consistency α coefficient, split reliability, and empirical validity of the scale were at 0.89, 0.86, and 0.66, respectively. The Cronbach’s α coefficient of this study is 0.952.

### Ethical consideration

The protocol for this study was approved by the ethics review committee of the Institutional Review Board (IRB) of Zhejiang University of Chinese Medicine (NO:20,200,507). To ensure anonymity, the identities, and names of the participants were not disclosed in this study. Participants gave informed consent, and were free to withdraw from the study without penalty.

### Data analysis

The IBM SPPS Statistics and AMOS Graphics packages were used for data analysis in this study. The results of the unary normality test showed that the absolute values of skewness, and kurtosis of the three variables are all within ± 2, indicating that the three variables conform to normal distribution [51]. Descriptive statistics were used to summarize the data on the demographic characteristics of the participants and their responses to daily stress, learning career adaptation, and mental health. The correlation analysis results among them were processed using Pearson’s correlation coefficients. AMOS 24.0 was used to construct a structural equation model to explore the mediating effect of learning career adaptation between daily stress, and mental health. A bootstrap method with deviation correction was used to test the significance of the mediating effect in AMOS 24.0.

## Results

### Common method biases test

A Harman single-factor test was performed to test for possible common methodological bias from self-reported data before data analysis [52]. Exploratory factor analysis was conducted on all items of the questionnaire. The results showed that there were 19 factors with characteristic roots greater than 1, and the explanation rate of the first common factor was 21.451%, much lower than 40%. Therefore, there is no significant common methodological bias in this study [53].

### Demographic characteristics of participants

Questionnaire network platform and convenience sampling were adopted in this survey, a total of 660 questionnaires were collected from 1593 medical-related postgraduate students in five universities, so the questionnaire recovery rate was 41.43%. After retrieving the questionnaires, the quality of the questionnaires was checked, we determined the effective questionnaire by eliminating too many differences between the average answer time, and the choices presented some patterns, such as zigzag answers. Twenty-five discarded questionnaires were destroyed, and the actual valid questionnaires totaled 635 with an effective rate of 96.21%. The valid questionnaire data were entered in time, and the data were checked by two people before and after the input. Of the 635 respondents, the ages were 25.11 ± 2.95, and 151 were male (23.78%), while 484 were female (76.22%). Those who majored in traditional Chinese medicine were at 158 (24.89%), clinical medicine at 245(38.58%), and nursing at 232 (36.54%). 255 (40.16%) were freshmen or first-year students, while sophomores totaled 244 (38.43%), and third-year students were 136 (21.42%). The demographic characteristics of participants are listed in Table 1.

**Table 1:** Socio-demographic characteristics of professional degree graduate students(n = 635)

	category	cases number	The proportion(%)
Gender	Male	151	23.78
	Female	484	76.22
The school	Zhejiang University Of Chinese medicine	232	36.54
	Fu Jian University Of Chinese medicine	63	9.92
	Hu Bei University of Chinese medicine	174	27.40
	Gui Yang University Of Chinese medicine	110	17.32
	Nan Jing University of Chinese medicine	56	8.82
Major	traditional Chinese medicine	158	24.89
	clinical medicine	245	38.58
	nursing	232	36.54
Grades	1st grade	255	40.16
	2nd grade	244	38.43
	3rd grade	136	21.42

### Descriptive statistics of mental health, daily stress, learning career adaptation

**Fig. 2:**
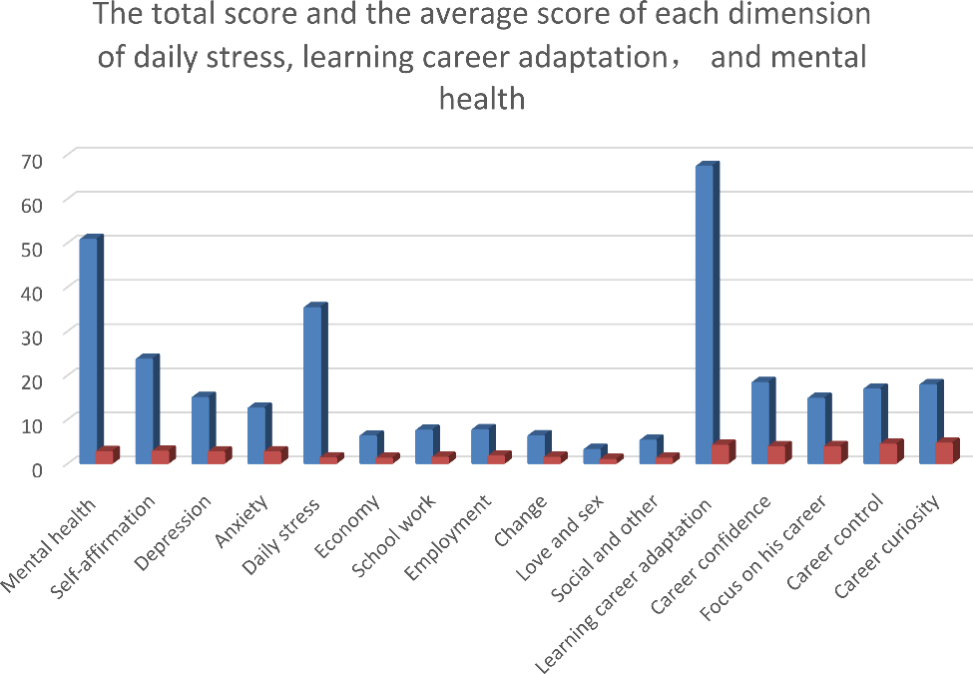
The total score, and the average score of each dimension of daily stress, learning career adaptation, and mental health

The total score of mental health was 50.56 ± 10.80(skewness = 0.228; kurtosis = -0.031). It scored from high to low on the dimensions of self-affirmation, anxiety, and depression. Daily stress was 35.12 ± 19.55 (skewness = 0.522; kurtosis = 0.112), with its dimensions scored in order of employment, school work, change, social and others, economy, and love and sex. The score of learning career adaptation was 67.13 ± 7.48 (skewness = − 1.086; kurtosis = 1.439), and its dimension scores were sorted as career curiosity, career control, focus on his career, and career confidence from high to low. The results of the descriptive statistics are shown in Table 2, and Fig. 2.

**Table 2:** The score of the questionnaire on mental health, daily stress, learning career adaptation(N = 635)

Variables	X ± S	Average
**Mental health**	50.56 ± 10.80	2.53 ± 0.54
Self-affirmation	23.44 ± 5.21	2.60 ± 0.58
Depression	14.77 ± 4.38	2.46 ± 0.73
Anxiety	12.35 ± 5.13	2.47 ± 1.02
**Daily stress**	35.12 ± 19.55	1.13 ± 0.63
Economy	6.07 ± 4.72	1.01 ± 0.79
School work	7.36 ± 4.46	1.23 ± 0.74
Employment	7.49 ± 4.45	1.50 ± 0.89
Change	6.11 ± 3.61	1.22 ± 0.72
Love and sex	3.01 ± 3.05	0.75 ± 0.76
Social and other	5.08 ± 3.65	1.02 ± 0.73
**Learning career adaptation**	67.13 ± 7.48	3.95 ± 0.44
Career confidence	18.16 ± 3.52	3.63 ± 0.70
Focus on his career	14.58 ± 2.83	3.65 ± 0.71
Career control	16.70 ± 1.84	4.18 ± 0.46
Career curiosity	17.68 ± 1.92	4.42 ± 0.48

### Correlation analysis of mental health, daily stress, and learning career adaptation

There was a remarkable negative correlation between daily stress, career confidence, focus on his career, and career control, which are the dimensions of learning career adaptation (the correlation coefficients were − 0.372, -0.338, and − 0.243, respectively, P<0.01). It was positively correlated with career curiosity (the correlation coefficient was 0.012), and the difference was not significant. Daily stress has a positive correlation with depression, and anxiety (correlation coefficient 0.232, and 0.418, P<0.01), and positively correlated with self-affirmation, but the difference was not significant. Self-affirmation was negatively correlated with career confidence, focus on his career (correlation coefficients were − 0.125, -0.116, P<0.05). Depression was negatively associated with career confidence, focus on his career, and career control (correlation coefficients were − 0.258, -0.233, and − 0.146, P<0.01). Anxiety was negatively associated with career confidence, focus on his career, and career control (correlation coefficients were − 0.404, -0.335, and − 0.238, P<0.01), but it had a positive relevance with career curiosity, no significant difference was found. The result of the correlation analysis is shown in Table 3.

**Table 3:** Correlation analysis of daily stress, learning career adaptation, and mental health

	1	2	3	4	5	6	7	8
**1**	1							
**2**	− 0.372^**^	1						
	0.000							
**3**	− 0.338^**^	0.736^**^	1					
	0.000	0.000						
**4**	− 0.243^**^	0.553^**^	0.546^**^	1				
	0.000	0.000	0.000					
**5**	0.012	-0.015	0.007	0.156^**^	1			
	0.792	0.745	0.882	0.001				
**6**	0.061	− 0.125^**^	− 0.116^*^	-0.041	0.088	1		
	0.196	0.008	0.014	0.387	0.063			
**7**	0.232^**^	− 0.258^**^	− 0.233^**^	− 0.146^**^	0.042	0.117^*^	1	
	0.000	0.000	0.000	0.002	0.373	0.013		
**8**	0.418^**^	− 0.404^**^	− 0.335^**^	− 0.238^**^	0.044	0.208^**^	0.588^**^	1
	0.000	0.000	0.000	0.000	0.354	0.000	0.000	

Note: 1 ~ 8 represent the Daily stress, Career confidence, Focus on his career, Career control, Career curiosity, Self-affirmation, Depression, Anxiety, respectively; * P < 0.05.** P < 0.01

### Mediating effect test

**Fig. 3:**
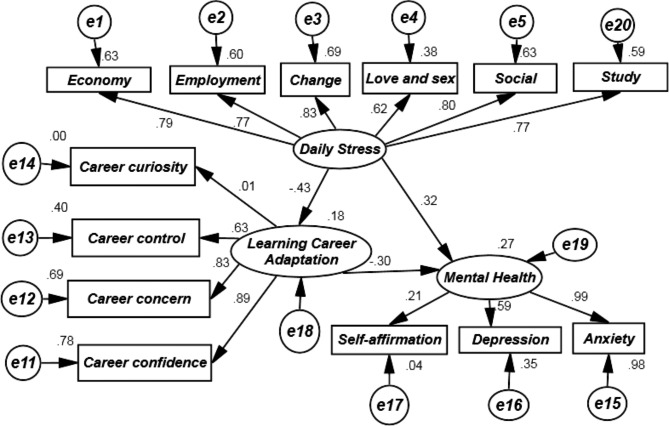
Analysis model results of the relationship between daily stress, learning career adaptation, and mental health

Gender, major, grades were controlled for the structural equations for each endogenous variable in the model. According to the hypothesis model, structural equation model parameter estimation, and mediation effect test were adopted, variance maximum likelihood method, and bootstrap test were used to test the mediation models, and a model with daily stress(six latent variables: economy, school work, employment, change, love and sex, social and other)as the independent variable, mental health as the dependent variable(three latent variables: self-affirmation, depression, anxiety), and learning career adaptation (four latent variables: career confidence, focus on his career, career control, career curiosity) as the mediating variable was established. The final model was obtained, as shown in Fig. 3. Each fitting index of the structural equation model meets the statistical requirements, and the model fit was good, as shown in Table 4. The results showed that daily stress directly affected learning career adaptation, and mental health, and the estimated values were − 0.427, and 0.318, respectively, the estimated value of learning career adaptation on mental health was − 0.298, and the differences were all statistically significant (P<0.01). Daily stress could indirectly affect mental health. The impact of daily stress on mental health, and its dimensions of self-affirmation, depression, and anxiety were 0.127, 0.093, 0.264, and 0.441, respectively, among which the impact on anxiety was the most obvious, and the indirect effect was significant (P<0.01). The estimated values of daily stress on learning career adaptation (career control, focus on his career, career confidence) were − 0.27, -0.355, and − 0.378, respectively, and the differences were significant ((P<0.01), among which daily stress had a more apparent impact on focus on his career and career confidence. The estimated values of learning career adaptation on self-affirmation, depression, and anxiety were − 0.063, -0.177, and − 0.296, respectively, and the differences were significant (P<0.01), among which the effect on anxiety was more obvious. Among the total effects, daily stress had significant effects on mental health, and learning career adaptation, and learning career adaptation had an obvious effect on mental health either, indicating that daily stress could directly affect mental health (effect size was 0.318), and daily stress could also affect mental health through its intermediary effect of learning career adaptation, and the effect value of this path was 0.127. Combining the two paths, the total predictive effect of daily stress on mental health was 0.445. Among them, the partial mediating effect of learning career adaptation accounted for 28.54% of the total effect, and the effect was significant. The deviation-corrected non-parametric percentage bootstrap (repeated sampling 2000 times) was used to test the mediating effect. The confidence intervals corresponding to each path of daily stress, and mental health did not contain 0, indicating that learning career adaptation played a partial mediating role in the relationship between daily stress, and mental health, as shown in Table 5.

**Table 4:** Structural equation model fitting index (n = 635)

Project	χ2	CMIN/df	RFI	NFI	CFI	IFI	TLI	RMSEA
**Modified fitting index**	146.013	2.355	0.926	0.941	0.965	0.965	0.956	0.055
**Acceptable standards**	/	< 5	> 0.9	> 0.9	> 0.9	> 0.9	> 0.9	< 0.08

Note: CMIN/df Maximum likelihood ratio χ2 values / degrees of freedom, RFI Relative fitness index, NFI Standard fit index, CFI Comparative fit index, IFI Value added index, TLI Nonstandard fit index, RMSEA Mean square sum square root of progressive residuals

**Table 5:** Analysis of the effect of daily stress, learning career adaptation on mental health

Effect	Estimated value	SE	Bootstrap		P value
			(95%CI)		
			lower boundaries	upper boundaries	
Direct effect					
DS-LCA	-0.427	0.065	-0.552	-0.294	0.001
DS-MH	0.318	0.052	0.213	0.421	0.001
LCA-MH	-0.298	0.061	-0.404	-0.203	0.001
Indirect effect					
DS-MH	0.127	0.031	0.075	0.196	0.001
DS-SA	0.093	0.032	0.037	0.161	0.001
DS-DP	0.264	0.041	0.190	0.346	0.001
DS-AX	0.441	0.043	0.353	0.522	0.001
DS-CCR	-0.006	0.021	-0.048	0.037	0.727
DS-CCT	-0.270	0.043	-0.358	-0.184	0.001
DS-FONC	-0.355	0.053	-0.457	-0.247	0.001
DS-CCF	-0.378	0.057	-0.487	-0.264	0.001
LCA-SA	-0.063	0.025	-0.121	-0.020	0.000
LCA-DP	-0.177	0.043	-0.271	-0.100	0.001
LCA-AX	-0.296	0.056	-0.407	-0.190	0.001
Total effect					
DS-LCA	-0.427	0.065	-0.552	-0.294	0.001
DS-MH	0.445	0.05	0.350	0.543	0.001
DS-SA	0.093	0.032	0.037	0.161	0.001
DS-DP	0.264	0.041	0.190	0.346	0.001
DS-AX	0.441	0.043	0.353	0.522	0.001
DS-CCR	-0.006	0.021	-0.048	0.037	0.727
DS-CCT	-0.27	0.043	-0.358	-0.184	0.001
DS-FONC	-0.355	0.053	-0.457	-0.247	0.001
DS-CCF	-0.378	0.057	-0.487	-0.264	0.001
LCA-MH	-0.298	0.061	-0.424	-0.183	0.001
LCA-SA	-0.063	0.025	-0.121	-0.020	0.000
LCA-DP	-0.177	0.043	-0.271	-0.100	0.001
LCA-AX	-0.296	0.056	-0.407	-0.190	0.001

Note: DS = Daily stress, LCA = Learning career adaptation, MH = Mental health, CCF = Career confidence, FONC = Focus on his career, CCT = Career control, CCR = Career curiosity, SA = Self-affirmation, DP = Depression, AX = Anxiety, respectively

## Discussion

The purpose of this study is to investigate the daily stress, learning career adaptation, and mental health of medical-related professional degree graduate students, and analyze the relationship among them, to provide a reference for improving their learning career adaptation, and mental health of them.

### Professional degree graduate students’ mental health is at a moderate degree

Scientists, psychologists, and educational researchers are concerned about the mental health of graduate students increasingly. They hope to investigate mental health further, hoping to find effective intervention measures to improve medical students’ quality of life [54]. The type of degree also affects the mental health of graduate students, with professional doctoral, and master’s students more likely to report mental health problems than academic graduate students [55]. This study found that the mental health average score was 2.53 ± 0.54, below the median value (3 points), it shows that the mental health level of professional degree graduate students is acceptable, and the subjects’ self-affirmation, and confidence were not enough, depression, and anxiety are more pronounced. Self-affirmation has a certain stress-buffering effect, and can improve the problem-solving ability of individuals with high stress [56]. It is suggested that educational managers should strengthen the self-esteem, and self-worth of professional degree graduate students, improve the acceptance of risk information to promote health behavior change, and pay close attention to their depression, anxiety, and other emotional changes. The outcomes of this study are similar to the results of Kondo’s study that Japanese students have a higher level of mental health compared with those in the United States [57]. Some studies have also found that graduate students have better mental health than undergraduates before and during the COVID-19 pandemic [58]. Considering maybe cultural differences, increasing ages, and maturity of mind play a vital role.

### Professional degree graduate students’ daily stress is to a lesser extent

Assessment of daily stress included: frequency, severity, associated emotions of stressors, permitted quantification of exposure, and cognitive evaluation of less stressful events in everyday life [22]. The average score of daily stress is 1.13 ± 0.63, which is below the median value of 2 points. Their daily lives stress reached an appropriate acceptable range, and showed that the most stressful things are jobs, and studies, with the least stress being the love and sex. Contrasting the results of Zhang [59] and other studies [60, 61], the psychological pressure of medical graduate students mostly came from economic pressure, followed by academic pressure, employment pressure, and future pressure. It’s also different from Rosiek’s study that everyday stressors included: study initiation, personal habits, changes in residence or diet, relationship difficulties with thesis supervisors or partners, and infections among medical students [62]. It showed that the current employment situation is severe for both medical, and nursing graduate students, including professional degree graduate students who have to focus on academic progress in heavy medical, and nursing work, especially for scientific research requirements, being tremendous sources of pressure for them. Therefore, educational managers should focus on evaluating the workload, and bearing capacity of professional degree graduate students. For students with learning difficulties, especially those with high pressure of scientific research, it should strengthen their care from the perspective of schools, and tutors, provide effective guidance, and help for individual students.

### Professional degree graduate students’ learning career adaptation is at an upper-middle level

Savickas redefined career adaptation as “the psycho-social construction of resource tasks, career changes, and personal trauma that individuals prepare for, and deal with current, and upcoming career development” [35]. In this study, the total average score of the learning career adaptation of professional degree graduate students was 3.95 ± 0.44. The scores of all factors were slightly higher than the medium critical value of 3 points, which was above the medium level, and consistent with the research results of clinical medical undergraduates [63], and the career curiosity score was the highest, and the career confidence score was the lowest. These outcomes indicated that graduate students of medical, and nursing should try, and explore actively, seek practical knowledge and use them passionately, and explore the suitable career path constantly for their future development. In terms of career confidence, most medical, and nursing graduate students lack sufficient confidence in their employment due to the tough employment situation.

### Correlations analysis of daily stress, learning career adaptation, and mental health in professional degree graduate students

It turned out that daily stress was positively correlated with mental health, verifying the first hypothesis: the more daily stress events occur, the lower the individual’s mental health will be. These results coincided with the findings of Xiu [21], and Zheng [64] that life events are positively correlated with depression, and anxiety [65]. Studies have also found that stress, psychological problems, and family status are the main factors affecting individual mental health [66]. Given high negative emotional responses to daily stressors, which are a strong predictor of future depression [26]. Sensitive emotional responses to daily stress may likewise lead to increased susceptibility to mental health disorders [67]. Interestingly, this study found that daily stress had a more positive predictive effect on anxiety, consistent with that some individuals showed higher levels of anxiety when daily stress increased [68]. Studies have found that the influence of anxiety state mainly depends on the intensity of stress, such as 5 min of pressure a day will not cause the change in anxiety state, and 10 min of pressure a day will significantly increase the level of anxiety [69], therefore, we should focus on the duration, and intensity of stressful events, being more sensitive to pressure, prone to the anxiety of professional degree graduate students’ mental health.

The study also found that daily stress was negatively correlated to learning career adaptation, proving the validity of the second hypothesis. These outcomes were associated with similar results of Zheng [64], and Zhang’s [70], in which that positive psychological resources such as resilience were negatively correlated with life events. Daily stress has a more significant negative effect on career confidence. Studies suggested that chronic stress can lead to burnout, which can lower career confidence, and even lead to quitting in turn [71]. Therefore, Compas [72] concluded that both primary, and secondary controlled participation coping are associated with adaptive mental health, including enhancement of positive emotions, and reduction of negative emotions, and effective coping occurs when the positive effects of coping or protective factors outweigh the negative effects of stressors [73], thus improved adaptation.

The study also found that learning career adaptation was related to mental health, thus also confirming the third hypothesis. Zhang [44] found a significant correlation that lies between the understanding of social support, career adaptation, and mental health of new graduate students. Studies have also shown that people with higher stress coping, and cross-cultural adaptation are more likely to have positive mental health disposition [74]. In the indirect effects of this study, it found that the negative effect of learning career adaptation on anxiety was more obvious. Chen’s research found that career planning can improve college graduates’ adaptability in uncertain situations, and markedly reduce their employment anxiety during the pandemic [75]. It was evident that learning career adaptation may be a protective factor of mental health in the case of low mental health caused by daily stress. Especially for some students who are prone to anxiety, it is more important to improve the adaptability of their learning career.

### Path analyses of the effects of daily stress, learning career adaptation on mental health in professional degree graduate students

This study also found that daily stress had a total effect, direct effect, and indirect effect on anxiety, and depression in mental health, while learning career adaptation played a partial mediating effect between daily stress, depression, and anxiety, proving the fourth hypothesis to be true. The results of this study showed that the path coefficient of daily stress on the learning career adaptation of professional degree graduate students (B = -0.427, p<0.001), and the path coefficient of learning career adaptation on mental health (B = -0.298, p<0.001), and the path coefficient of daily stress on mental health (B = 0.445, p<0.001). The learning career adaptation had a significant, and partial mediating effect between daily stress, and mental health of professional degree graduate students, and the mediating value was 0.127. Through the interaction between the individual, and his living environment, career adaptation becomes a plasticity resource to improve an individual’s subjective feelings, and well-being [76, 77]. In the increasingly unstable working environment, students’ career adaptation is essential in the future [78]. Many scholars believe that students need to develop positive career adaptation to cope with changing occupational tasks, and environments [79, 80].

### How to improve professional degree graduate students ' learning career adaptation, and mental health?

Although graduate students are more mature, and independent than college students, they will suffer from psychological imbalance, and psychological crisis if the pressure, and conflict between family responsibilities, social expectations, economic pressure, and career achievements are not handled properly [20]. Professional degree graduate students face more pressure in study, and work, so it is urgent to improve their mental health. Firstly, assessment, and screening are the first step to promote mental health. In addition to screening, psychological census filing, lectures, and crisis intervention should be included in the teaching system, and the curriculum platform for graduate students’ mental health education should be developed. Secondly, Professional degree graduate students are encouraged to take part in sports actively. Low-dosed, short-duration physical activity interventions, and cognitive interventions consisting of positive expressive writing can buffer students’ stress, mood, and quality of life [81]. It was proved that low-to-moderate-intensity aerobic exercise for six weeks was able to attend to act as a buffer against depression, and perceived stress [82]. Thirdly, providing interesting courses, and paying more attention to personal characteristics are conducive to maintaining mental health. A study found that the key to improving the mental health of freshmen is to provide a wide range of courses, enhance their adaptive ability, and pay attention to their demographic characteristics, and cognitive characteristics [83]. Mental health education activities should be carried out through various activity carriers vigorously, and the psychological support system, including network media, should be established, and played an important role [84]. Mental health first aid e-learning courses have the potential to improve first aid skills of mental health, confidence in helping friends, and stigmatizing attitudes among UK medical students. It may be useful to support one’s own, and others’ mental health during study, and in future healthcare careers [85]. Fourthly, effective social support is conducive to mental health. Positive coping styles, and social support can improve the mental health of graduate students, and alleviate abnormal psychological symptoms through the gain effect [86]. Therefore, Professional degree graduate students should be provided with material, mental, information, and other support in time when they encounter studying, and working pressure. Fifth, the application of interventions that could promote mental health, such as internet-based mindfulness training, and cognitive behavior training, can improve mental health, life satisfaction, adjust sleep disorders, and psychological distress of college students after three months of trying [87]. In addition, Van Agteren [88] found that the mapping approach intervention could promote mental health through case studies. Mental health education based on music therapy was also considered to be an effective way [89]. During the COVID-19 lockdown, a Canadian study identified a need for increased mental health education awareness, and mental health resources in postsecondary institutions, as well as support for institutional, and mental health services [90]. Improving the availability of mental health resources within schools to increase accessibility and using of these services for all students, emphasizing the importance of fairness [91]. Many college students have barriers in receiving in-person mental health help due to concerns about stigma of mental health problems, but most of them find in-person therapy, and online care by therapists more helpful than self-directed online care [90]. Therefore, many higher education institutions in Europe, and the United States provide virtual counseling services, and general psychological education resources during the epidemic period [92], [93]. That is, making full use of some APP programs of smart phones, such as TIKTOK, to provide students with instant, and convenient mental health educational services. For example, the University of Texas has announced that it will expand its use of mental health-oriented smartphone applications over the next five years in 2022 [94].

Career adaptation emphasizes that individuals can explore, and construct their values, and abilities in specific activities, and experiences by establishing positive relations with the subjective, and objective world [95]. It is the result of the interaction between individuals, and the environment, which can be cultivated, and developed subsequently [37]. Professional degree graduate students were familiar with the content, and process of the clinical practice through the undergraduate internship period relatively. However, the input to the internship practice was limited due to preparation for the postgraduate entrance examination. They have required standardized training in the clinical practice period of professional degree graduate students, and the workload, responsibility, theoretical knowledge, professional skills, and new professional progress requirements are all higher than before. Moreover, it is difficult to find, and solve clinical problems from the perspective of scientific research for them. Most of the undergraduate students did not have scientific research experience, so the professional degree graduate students are very anxious at the beginning of their study, so it is necessary to help them to enhance learning career adaptation. Nevertheless, there are limited empirical studies on the improvement of learning career adaptation, this study believes that the learning career adaptation of professional degree graduate students might be improved from the perspective of organizations, and individuals.

The first is from an organizational perspective, professional assessment tools could be used in school to help students understand their interests, characters, abilities, and values, such as the MBTI personality test, and Holland career interest test, as well as gain more information about their career situation, development path, and rewards. Understanding who I am (self-exploration), what my options are (career world exploration), and where I want to go (career decisions). Then, the school can set up a career file for each professional degree graduate student, which should be perfected, and kept by the students themselves. The teacher of career education should check it, and guide them regularly. The career files include career exploration, practical training records, social practice activity records, career training records, internship reports, and other aspects, which can be used as support, and proof materials for job hunting. In making, and perfecting the files, the students are urged to complete career concern, and career control. It should also strengthen the career adaptation counseling courses to guide students to understand the current situation, development trend, professional knowledge, and skills required by the job, and accept the uncertainty of the career which helps individuals better adapt to the changes of employment, and social environment to improve their career curiosity. Students are encouraged to participate in career planning, and career design competitions actively, master the skills of career planning, improve their understanding, and recognition of their professional tasks, and build students’ career confidence [96]. Teachers should guide students’ study, and life, interpersonal communication, emotion management, and time management. The university can provide training, and consulting services in statistics, mapping, animal model building, and so on for professional degree graduate students. We should also cultivate the ability of self-regulated learning, and develop their professional abilities according to the needs of professional development. Career counseling studios can be set up where psychological or career counselors could provide personalized counseling for students with career confusion, help them strengthen their career control, and relieve the pressure of employment, and entrepreneurship. For example, the 8-week career group counseling intervention based on Savickas theory can improve the career adaptation of new nurses [97]. Furthermore, individuals with high adversity quotient are more likely to turn obstacles into opportunities in their careers, which can promote an individual^’^s career adaptation [98]. The grit training based on a self-regulation process can improve career adaptation [99]. Therefore, it should strengthen frustration education, and enhance resilience, psychological capital, and other psychological resources education in universities. In addition to this, it found that the learning goal orientation, supervisor incompetence accusations, as well as career development training can promote career adaptation through the mediating role of deliberate practice of their professional activities [100].

At the same time, the management rules of the tutorial system should be formulated to ensure regular communication between tutors, and students from the perspective of hospital management. The managers should standardize the management of the tutorial system, hold regular team meetings, carry out academic exchanges, and reports, and ensure a certain amount of scientific research work time for professional degree graduate students. The students may have a corresponding understanding of the required qualifications, job nature, and content, work rhythm, occupational skill intensity, work environment, work salary, future development prospects, and other content utilizing practitioners’ lectures and hospital practice to improve their career concern, and career curiosity. The professional theory, and skills of students can be enhanced through lectures, ward rounds, professional skills competitions, and examinations in the hospital. So that the students can apply what they have learned to obtain a successful experience fully, or invite graduated seniors to give lectures to increase career confidence. In addition, the hospital can also hold speech contests, and communicational case analysis meetings to exercise students’ eloquence, and interpersonal communication skills. Finally, mock job fairs can be held to examine students’ career preparation.

The second is from a personal perspective, some studies have found that there is a correlation between career adaptation, and personality traits. Openness, and conscientiousness can promote career adaptation, while the other three personality types have no significant effect on it [101]. Therefore, individuals should adapt to the pressure, and stress of study, scientific research, clinical practice, and employment zealously, and cope with them actively. Individuals should not only improve their knowledge, and skills according to the ability, and requirements of their future career, but also strengthen their interpersonal communication ability according to the characteristics of the medical profession after selecting the direction of their career development. Moreover, students are encouraged to attribute success, and failure correctly, and have a good sense of autonomy, and responsibility to adapt to the environment, to improve career control.

### Strengths and contributions of the current research

The theoretical contributions of this study are as follows: (1) this study conducted a relatively complete analysis of the relationship between daily stress, and internal (learning career adaptation) and external (daily stress) indicators of mental health, enriching the exploration of influencing factors of mental health. (2) This study explored the relationship between daily stress, and mental health, and established a theoretical framework of mental health mediated by learning career adaptation based on social adaptation, which provided a new perspective for in-depth analysis of the formation path of mental health. The enlightenment to practice is that the mental health of an individual is affected by both external, and internal factors. (1) Daily stress has a significant predictive effect on mental health. As for individuals, they should give full play to their psychological resources, and realize positive adaptation in the secondary evaluation of stress. In the evaluation of students’ mental health, the school should pay attention to screening the students with more significant stress load, and conducts dynamic observation on them. (2) The mediating effect of learning career adaptation also suggested that educational managers should focus on the cultivation of learning career adaptation of professional degree graduate students. From the perspective of individuals, and organizations (schools, and hospitals), the learning career adaptation of students can be improved from four aspects: career confidence, focus on his career, career control, and career curiosity.

### Limitations

There are certain limitations associated with any study. First, convenience sampling, and low recovery rates (Considered as the students were busy to work, and study, and didn’t have the time to complete the questionnaire), which led to the study results overgeneralization possibly. It should be noted that the disciplines are professional degree graduate students in traditional Chinese medicine, clinical medicine, and nursing. The sample sources, and regions were relatively limited, the self-report questionnaire was used, and there was no objective index. Future researchers need to be careful about extrapolating to other schools, professionals, and genders if the outcomes of the complex relationships between mental health, daily stress, and learning career adaptation are to be explored. It can consider longitudinal study design, or the simultaneous measurement of multiple variables at multiple time points in the future to investigate the changes of variables, and the influence relationship between variables. The research team will explore other factors that affect the mental health of professional degree graduate students deeply, and explore the practical effects of the intervention actively in the future.

## Conclusions

This study revealed the effect of daily stress (external factors) on the mental health of professional degree graduate students in medical-related majors in traditional Chinese medicine universities, and explored the partial mediating role of learning career adaptation (internal psychological resources) in the daily stress, and mental health of professional degree graduate students, which is of great significance to expand, and deepen the psychological, cognitive, and behavioral responses to stress adaptation theory. It provides a resource perspective for the educational management of professional degree graduate students in medical-related majors, which can improve students’ learning career adaptation, and mental health from the individual, and organizational perspectives.

### Electronic supplementary material

Below is the link to the electronic supplementary material.

Additional file 1: English version of the questionnaire

## Data Availability

The datasets used and/or analyzed during the current study are in Chinese and are available from the corresponding author on reasonable request but will require translation to English.
