# Supplementary material for: Daily stress, and mental health of professional degree graduate students in Chinese traditional medicine universities: the mediating role of learning career adaptation
Source: BMC Med Educ. 2023 Sep 3;23:627. doi: 10.1186/s12909-023-04614-5 (PMC10476438; doi:10.1186/s12909-023-04614-5)
Supplement: Supplementary file 1 — Additional file 1: English version of the questionnaire [file 12909_2023_4614_MOESM1_ESM.docx]

Dear students:

Hello! This is a questionnaire about learning adaptation and mental health of professional master students, the purpose of the survey is to understand the basic situation of mental health of contemporary professional master students. Your answers will provide valuable basis for our research, and will also provide important help for professional master students' learning! Please take some precious time to read the questions carefully and then answer according to your actual situation! The questionnaire does not need to be named, the answer is no right or wrong, only for research use, We will keep your answers absolutely confidential! Therefore, you do not need to have any concerns, please feel free to answer! Thank you for your cooperation!

Please mark "√" in the corresponding "□" according to the degree that each sentence is in line with you. Choose only one answer for each question. Please do not choose more than one or miss the selection.

Questionnaire 1: Graduate students learning career adaptation Scale

| project | | Completely inconsistent | Inconsistent | Uncertain | Consistent | Completely consistent |
| --- | --- | --- | --- | --- | --- | --- |
| 1 | I will try a variety of ways (taking uizzes/counseling/working) to explore my values, interests, and abilities. | 1 | 2 | 3 | 4 | 5 |
| 2 | In the face of setbacks, I always see the positive meaning behind the setbacks. | 1 | 2 | 3 | 4 | 5 |
| 3 | Although the future is unknown, I think I can control the future direction of career development. | 1 | 2 | 3 | 4 | 5 |
| 4 | During my postgraduate period, I not only paid attention to the improvement of academic level, but also paid attention to the cultivation of my interests. | 1 | 2 | 3 | 4 | 5 |
| 5 | After setbacks and failures, I will reflect on myself and listen to others. | 1 | 2 | 3 | 4 | 5 |
| 6 | During my postgraduate period, I often participated in various social practice activities to cultivate my practical ability. | 1 | 2 | 3 | 4 | 5 |
| 7 | Every time I go to a new place, it is always easier for me to establish a good relationship with others. | 1 | 2 | 3 | 4 | 5 |
| 8 | I believe that I have strong working ability and can do my job well. | 1 | 2 | 3 | 4 | 5 |
| 9 | I often consciously pay attention to the development prospects and social application of this major. | 1 | 2 | 3 | 4 | 5 |
| 10 | For the future, I have a clear goal and direction in mind. | 1 | 2 | 3 | 4 | 5 |
| 11 | I attach great importance to the relationship between what I have done and learned and my future career development. | 1 | 2 | 3 | 4 | 5 |
| 12 | I can make my career choices independently, even without the approval and support of others. | 1 | 2 | 3 | 4 | 5 |
| 13 | I believe I can perform well in all kinds of environments. | 1 | 2 | 3 | 4 | 5 |
| 14 | I think I have a good adaptability and can successfully navigate various career transitions. | 1 | 2 | 3 | 4 | 5 |
| 15 | In interpersonal communication, I know how to get along well with others. | 1 | 2 | 3 | 4 | 5 |
| 16 | No matter what happens in my future career, I will take responsibility for my career choice. | 1 | 2 | 3 | 4 | 5 |
| 17 | I like to think about my future career development and direction, and I am full of hope for the future. | 1 | 2 | 3 | 4 | 5 |

Questionnaire 2: Daily stressors scale for graduate students

|  | **project** | No impact | **Mild** | Moderate | **Severe** | **Extremely severe** |
| --- | --- | --- | --- | --- | --- | --- |
|  |  | 0 | 1 | 2 | 3 | 4 |
| 1 | Study content or specialty is boring | 0 | 1 | 2 | 3 | 4 |
| 2 | Dissatisfied with the school's training model and curriculum | 0 | 1 | 2 | 3 | 4 |
| 3 | The project is not going well | 0 | 1 | 2 | 3 | 4 |
| 4 | Others expect more of themselves than ever before | 0 | 1 | 2 | 3 | 4 |
| 5 | Daily expenses lead to financial stress | 0 | 1 | 2 | 3 | 4 |
| 6 | There was a large variation in the type or number of social activities | 0 | 1 | 2 | 3 | 4 |
| 7 | To be misunderstood or wronged | 0 | 1 | 2 | 3 | 4 |
| 8 | Tuition pressure | 0 | 1 | 2 | 3 | 4 |
| 9 | Major changes in personal lifestyle (e.g., diet, sleep) | 0 | 1 | 2 | 3 | 4 |
| 10 | Major is not good; employment prospects are unfavorable | 0 | 1 | 2 | 3 | 4 |
| 11 | The family economic situation is not good | 0 | 1 | 2 | 3 | 4 |
| 12 | Part-time or assistant job is not ideal | 0 | 1 | 2 | 3 | 4 |
| 13 | Great pressure on employment | 0 | 1 | 2 | 3 | 4 |
| 14 | Scholarships or other awards become unavailable | 0 | 1 | 2 | 3 | 4 |
| 15 | Have to participate in certain social activities | 0 | 1 | 2 | 3 | 4 |
| 16 | In love or out of love | 0 | 1 | 2 | 3 | 4 |
| 17 | Personal lending | 0 | 1 | 2 | 3 | 4 |
| 18 | Significant changes in learning styles | 0 | 1 | 2 | 3 | 4 |
| 19 | Have trouble with your lover | 0 | 1 | 2 | 3 | 4 |
| 20 | Some classes are boring, but you have to take them | 0 | 1 | 2 | 3 | 4 |
| 21 | A significant change in one's values or self-evaluation | 0 | 1 | 2 | 3 | 4 |
| 22 | Social competition is becoming increasingly fierce, worried about employment | 0 | 1 | 2 | 3 | 4 |
| 23 | Illness or injury | 0 | 1 | 2 | 3 | 4 |
| 24 | Sexual distress | 0 | 1 | 2 | 3 | 4 |
| 25 | Worry about their ability is not strong that affect employment | 0 | 1 | 2 | 3 | 4 |
| 26 | Conflict with others | 0 | 1 | 2 | 3 | 4 |
| 27 | Worry related to the publication of papers | 0 | 1 | 2 | 3 | 4 |
| 28 | A relative or friend is in trouble | 0 | 1 | 2 | 3 | 4 |
| 29 | The confusion about finding a male (female) friend | 0 | 1 | 2 | 3 | 4 |
| 30 | In contrast, the laboratory conditions are not good | 0 | 1 | 2 | 3 | 4 |
| 31 | Lack of topics or poor guidance from tutors | 0 | 1 | 2 | 3 | 4 |

Questionnaire 3: Psychological Resilience Questionnaire for University Students

| **serial number** | **title** | **Never** | **Seldom** | **Sometimes** | **Frequently** | **Always** |
| --- | --- | --- | --- | --- | --- | --- |
| 1 | I can adapt to change | 0 | 1 | 2 | 3 | 4 |
| 2 | I have a close, secure relationship | 0 | 1 | 2 | 3 | 4 |
| 3 | Sometimes, fate or God can help | 0 | 1 | 2 | 3 | 4 |
| 4 | I can handle whatever happens | 0 | 1 | 2 | 3 | 4 |
| 5 | My past success gives me the confidence to face the challenge | 0 | 1 | 2 | 3 | 4 |
| 6 | I can see the humorous side of things | 0 | 1 | 2 | 3 | 4 |
| 7 | Coping with stress makes me feel empowered | 0 | 1 | 2 | 3 | 4 |
| 8 | I tend to recover quickly from hardship or illness | 0 | 1 | 2 | 3 | 4 |
| 9 | Things happen for a reason. | 0 | 1 | 2 | 3 | 4 |
| 10 | Whatever the outcome, I'll do my best. | 0 | 1 | 2 | 3 | 4 |
| 11 | I can achieve my goals | 0 | 1 | 2 | 3 | 4 |
| 12 | I don't give up easily when things seem hopeless | 0 | 1 | 2 | 3 | 4 |
| 13 | I know where to go for help | 0 | 1 | 2 | 3 | 4 |
| 14 | I can concentrate and think clearly under pressure | 0 | 1 | 2 | 3 | 4 |
| 15 | I like to take the lead in solving problems | 0 | 1 | 2 | 3 | 4 |
| 16 | I will not be discouraged by failure | 0 | 1 | 2 | 3 | 4 |
| 17 | I consider myself a strong person | 0 | 1 | 2 | 3 | 4 |
| 18 | I can make unusual or difficult decisions | 0 | 1 | 2 | 3 | 4 |
| 19 | I can deal with unhappiness. | 0 | 1 | 2 | 3 | 4 |
| 20 | I had to act on my hunch | 0 | 1 | 2 | 3 | 4 |
| 21 | I have a strong sense of purpose | 0 | 1 | 2 | 3 | 4 |
| 22 | I feel in control of my life. | 0 | 1 | 2 | 3 | 4 |
| 23 | I like a challenge. | 0 | 1 | 2 | 3 | 4 |
| 24 | I work hard to achieve my goal | 0 | 1 | 2 | 3 | 4 |
| 25 | I am proud of my achievements | 0 | 1 | 2 | 3 | 4 |

Questionnaire 4: General Health Questionnaire (GHQ-20)

| **serial number** | **title** | Completely does not comply | Less complies | Uncertain | Mostly complies | Fully complies |
| --- | --- | --- | --- | --- | --- | --- |
| 1 | In general, I'm happy with everything. | 1 | 2 | 3 | 4 | 5 |
| 2 | If you do things to be able to concentrate | 1 | 2 | 3 | 4 | 5 |
| 3 | Whether very satisfied with his way of doing things | 1 | 2 | 3 | 4 | 5 |
| 4 | Have you been busy lately and making good use of your time | 1 | 2 | 3 | 4 | 5 |
| 5 | Manage the daily affairs is as good as that of others | 1 | 2 | 3 | 4 | 5 |
| 6 | Feel yourself on a lot of things can help or provide some opinions | 1 | 2 | 3 | 4 | 5 |
| 7 | Feeling very unhappy and depressed | 1 | 2 | 3 | 4 | 5 |
| 8 | Be able to live your normal life happily | 1 | 2 | 3 | 4 | 5 |
| 9 | Is it easy to get along with people | 1 | 2 | 3 | 4 | 5 |
| 10 | Feel there is hope in the future | 1 | 2 | 3 | 4 | 5 |
| 11 | Think to be not what do you mean | 1 | 2 | 3 | 4 | 5 |
| 12 | Lose confidence in myself | 1 | 2 | 3 | 4 | 5 |
| 13 | Feel that life is completely hopeless | 1 | 2 | 3 | 4 | 5 |
| 14 | I feel like a useless person | 1 | 2 | 3 | 4 | 5 |
| 15 | Life feels like a war zone all day long | 1 | 2 | 3 | 4 | 5 |
| 16 | You can't sleep because you're worried | 1 | 2 | 3 | 4 | 5 |
| 17 | Is the mood upset sleep bad | 1 | 2 | 3 | 4 | 5 |
| 18 | Feel uneasy and nervous all day long | 1 | 2 | 3 | 4 | 5 |
| 19 | Do you feel stressed all day | 1 | 2 | 3 | 4 | 5 |
| 20 | Because feel too nervous sometimes can't do anything | 1 | 2 | 3 | 4 | 5 |

Questionnaire 5：Demographic characteristics data

| Gender： |  | Age： |  | | Research Directions： | |  | | Training hospital**：** |  |
| --- | --- | --- | --- | --- | --- | --- | --- | --- | --- | --- |
| **Nature of Master's Degree** | | | | | | | | | | |
| A. Academic degree | | | | B. Professional degree | | | |  | | |
| **Research Classification** | | | | | | | | | | |
| A. Master of Chinese Medicine | | | | B. Master of Western medicine | | | | C. Nursing | | |
| **Graduate school form** | | | | | |  | | | | |
| A. On the job | | | | B. off the job | | | |  | | |
| **Nature of tariff** | | | |  | | | |  | | |
| A. Publicly funded | | | | B. Self-financing | | | | C. Commissioning | | |
| **Whether to change major to graduate school** | | | | | | | |  | | |
| A.Yes | | | | B. No | | | |  | | |
| **Grades** | | | |  | | | |  | | |
| A. Grade 1 | | | | B. Grade 2 | | | | C. Grade 3 | | |
| **Source of students** | | | |  | | | |  | | |
| A. Cities | | | | B. Rural | | | | C. Other | | |
| **Years of service** | | | |  | | | |  | | |
| A. No | | | | B.1-2.9 years | | | | C.3-4.9 years D. More than 5 years | | |
| **Years of standardized training** | | | |  | | | |  | | |
| A. No | | | | B. One year | | | | C. Two years D. Three years | | |
| **Won a scholarship last semester** | | | |  | | | |  | | |
| A. obtained | | | | B. not obtained | | | |  | | |
| **Whether for student loans** | | | |  | | | |  | | |
| A.Yes | | | | B. No | | | |  | | |
| **Financial situation (cost of living)** | | | | | | | |  | | |
| A good | | | | B. General | | | | C. Not good | | |
| **Social support situation** | | | |  | | | |  | | |
| A good | | | | B. General | | | | C. Not good | | |
| **Serve as a student cadre?** | | | |  | | | |  | | |
| A.Yes | | | | B. No | | | |  | | |
| **Your grades are ranked in the class** | | | | | | | |  | | |
| A. Upstream | | | | B. Midstream | | | | C. Downstream | | |
| **Do you like your major？** | | | | | | | |  | | |
| A. Like | | | | B. General | | | | C. Not good | | |
| **If there is a scientific research experience before** | | | | | | | |  | | |
| A.Yes | | | | B. No | | | |  | | |
| **Whether you feel pressure to write the paper or dissertation** | | | | | | | |  | | |
| A.No | | | | B. General | | | | C. large | | |
| **Whether for clinical practice or clinical practice in the future and feel the pressure?** | | | | | | | | | | |
| A.No | | | | B. General | | | | C. large | | |
| **Whether you feel pressure to communicate with your tutor?** | | | | | | | | | | |
| A.No | | | | B. General | | | | C. large | | |
| **Whether you feel pressure to communicate with the patient?** | | | | | | | | | | |
| A.No | | | | B. General | | | | C. large | | |
| **Whether or not you are an only child** | | | | | | | |  | | |
| A.Yes | | | | B.No | | | |  | | |
| **Marital status** | | | |  | | | |  | | |
| A.Married . | | | | B.Unmarried | | | | C.Divorce | | |
| **Children number** | | | |  | | | |  | | |
| A.No | | | | B. one | | | | C.2 or more | | |
